# The *Riemerella anatipestifer M949_RS01035* gene is involved in bacterial lipopolysaccharide biosynthesis

**DOI:** 10.1186/s13567-018-0589-8

**Published:** 2018-09-17

**Authors:** Yafeng Dou, Guijing Yu, Xiaolan Wang, Shaohui Wang, Tao Li, Mingxing Tian, Jingjing Qi, Chan Ding, Shengqing Yu

**Affiliations:** 1 Shanghai Veterinary Research Institute, Chinese Academy of Agricultural Sciences (CAAS), Shanghai, China; 2 Jiangsu Co-innovation Center for Prevention and Control of Important Animal Infectious Diseases and Zoonosis, Yangzhou, People’s Republic of China

## Abstract

In this study, the *Riemerella anatipestifer* mutant strain RA1062 was obtained by screening a random Tn4351 transposon mutant library. The mutant strain was unreactive with the anti-CH3 lipopolysaccharide monoclonal antibody, as demonstrated with an enzyme-linked immunosorbent assay, and its *M949_RS01035* gene was inactivated. When cultured in trypticase soy broth, the late stage growth of the mutant RA1062 was significantly decreased. The mutant RA1062 was stained with crystal violet and presented a rough lipopolysaccharide phenotype, which differed from that of the wild-type strain CH3, suggesting that deletion of the *M949_RS01035* gene resulted in defective lipopolysaccharide. Silver staining and Western blot analyses further confirmed that the RA1062 lipopolysaccharide had a deficiency in ladder-like binding pattern, as compared to lipopolysaccharide of the wild-type CH3 strain. In addition, the mutant RA1062 showed a higher susceptibility to complement-dependent killing, increased bacterial adhesion and invasion capacities to Vero cells, decreased blood bacterial loads, and attenuated virulence in infected ducks, when compared to the wild-type strain CH3. Moreover, RNA-Seq and real-time polymerase chain reaction analyses indicated that two genes were up-regulated and two were down-regulated in the mutant RA1062 genome. Furthermore, an animal protection experiment showed that immunization of ducks with inactivated RA1062 bacterin conferred effective cross-protection against challenge with the virulent *R. anatipestifer* serotypes 1, 2, and 10. This study presents evidence that the *M949_RS01035* gene is involved in bacterial phenotype, virulence, and gene regulation in *R. anatipestifer*. The mutant strain RA1062 could be used as a cross-protective vaccine candidate.

**Electronic supplementary material:**

The online version of this article (10.1186/s13567-018-0589-8) contains supplementary material, which is available to authorized users.

## Introduction

*Riemerella anatipestifer* is a Gram-negative, non-motile, non-spore forming, and atrichous bacterium belonging to the family *Flavobacteriaceae* in the phylum *Bacteroidetes* [1]. *R. anatipestifer* has received considerable attention because infection of this bacterium cause major economic losses to the duck farming industry through high mortality, weight loss, and high treatment costs [2]. *R. anatipestifer* infection is a highly contagious disease that causes fibrinous pericarditis, airsacculitis, and perihepatitis in ducks [3]. To date, 21 *R. anatipestifer* serotypes have been identified worldwide; however, there is currently no effective cross-protection between different serotypes [4, 5]. In China, serotypes 1, 2, and 10 are responsible for most outbreaks of *R*. *anatipestifer* infection [6].

A variety of vaccines to protect farm ducks from *R. anatipestifer* infection have been investigated, including the chaperonin GroEL and inactivated bacterin [7, 8]. On the other hand, some potential virulence factors of *R. anatipestifer* have been described, including OmpA, VapD, and CAMP cohemolysin [9, 10].

As with other Gram-negative bacteria, lipopolysaccharide (LPS) is probably one of the most important virulence factors of *R*. *anatipestifer*. LPS is the major outer membrane component of Gram-negative bacteria and typically comprise three structure domains: lipid A, a core oligosaccharide, and a polysaccharide O-antigen. The O-antigen is composed of repeated sequences of three to six sugar residues and is also the most structurally variable cell surface constituent. Pathogenic bacterial O-antigen plays important roles in avoiding phagocytosis and resisting the lytic action of the complement system [11–14]. The O-antigen has been also a basis for vaccine development against a variety of human pathogens, [15]. In several species of bacteria, such as *Escherichia coli* and *Salmonella enterica*, the genes involved in LPS biogenesis have been well characterized [16, 17]. In *R. anatipestifer*, five genes (*AS87_04050*, *M949_1556*, *M949_1603*, *M949_1360* and *M949_RS01915*) associated with LPS synthesis have been characterized in our previous studies [18–22].

In this report, we described the identification of the *R. anatipestifer* mutant strain RA1062, in which the *M949_RS01035* gene was disrupted by insertion of the Tn4351 transposon. Furthermore, the LPS phenotype, bacterial virulence, gene regulation, and the cross-protection of the mutant strain RA1062 were characterized.

## Materials and methods

### Ethics statement

The study protocol was approved by the Institutional Animal Care and Use Committee of Shanghai Veterinary Research Institute, the Chinese Academy of Agricultural Sciences (Approval No. Shvri-po-0072), and conducted in strict accordance with the recommendations outlined in the Guide for the Care and Use of Laboratory Animals. One-day old Cherry Valley ducks were obtained from Zhuang Hang Duck Farm (Shanghai, China) and housed in cages under a controlled temperature of 28–30 °C with water and food ad libitum under biosafety conditions.

### Bacterial strains and growth conditions

The bacterial strains and plasmids used in this study are listed in Table 3. The mutant strain RA1062 was derived from the wild-type (WT) *R. anatipestifer* strain CH3 (GenBank accession no. CP006649), which is a serotype 1 strain, by insertion of the Tn4351 plasmid. *R. anatipestifer* strains were grown on tryptic soy agar (TSA; Difco Laboratories, Franklin Lakes, NJ, USA) or in tryptic soy broth (TSB, Difco) at 37 °C under an atmosphere of 5% CO_2_.

*Escherichia coli* strains were grown at 37 °C on Luria–Bertani (LB) plates or in LB broth. When necessary, antibiotics were added to the medium at the following concentrations: kanamycin at 50 μg/mL and erythromycin at 0.5 μg/mL for the mutant strain RA1062, and chloramphenicol at 30 μg/mL for *E. coli* strain BW19851 (pEP*4351*).

### Indirect enzyme-linked immunosorbent assay (ELISA)

An indirect ELISA was used to screen the Tn4351 insertion mutant library for strains with defective reactivity to the anti-CH3 LPS monoclonal antibody (mAb), as described previously [18]. Briefly, each well of a 96-well ELISA plate was coated with whole mutant cells at 10^9^ CFU/well in 50 μL of carbonate-buffered saline (pH 9.6). The plates were heat-dried overnight at 55 °C. After washing three times with phosphate-buffered saline (PBS) containing 0.05% Tween 20 (PBST), the plates were blocked with PBS-5% skim milk at 37 °C for 2 h, then incubated with anti-CH3 LPS mAb, as the primary antibody, and horseradish peroxidase-conjugated anti-mouse immunoglobulin G (IgG; Tiangen Biotech (Beijing) Co., Ltd., Beijing, China), as the secondary antibody. The reaction was visualized by adding 100 μL of 3,30,5,50-tetramethyl benzidine (Tiangen) and stopped by the addition of 50 μL of 2 M H_2_SO_4_. The resulting optical density at 450 nm (OD_450_) was measured using a plate reader (Synergy 2; BioTek Instruments, Inc., Winooski, VT, USA). The WT strain CH3 was used as a positive coating control. The mutants with its coating wells presenting an OD_450_ reading more than 2.1 times lower than that of the positive coating control were collected for further characterization. All mutants were screened in triplicate.

### Characterization of the mutant strain RA1062

The WT strain CH3 and the mutant strain RA1062 were identified by polymerase chain reaction (PCR) analysis with the primers 16S rRNA F/16S rRNA R, Erm-F/Erm-R, and RA1062-F/RA1062-R (Table 1). Bacterial mutants were constructed by transposon mutagenesis as described previously [23], with modifications. The *E. coli* BW19851 with the plasmid pEP4351 was used as the donor strain and *R. anatipestifer* CH3 as the recipient. For bacterial mating, both donor and recipient bacteria were grown to mid-logarithmic phase, mixed at a bacterial colony forming units (CFU) ratio of 1:2 and centrifuged at 5500 × *g* for 10 min. The bacterial pellet was washed and re-suspended with 10 mM MgSO_4_, and filtered through a Millipore membrane, which was then placed face up on TSA with 1 μg/mL erythromycin and 50 μg/mL kanamycin. Following overnight incubation at 30 °C, the bacteria were scraped off the filter, resuspended in 5 mL 10 mM MgSO_4_, and spread on TSA containing erythromycin and kanamycin to select for transconjugants. To confirm one insertion of the Tn4351 transposon in the mutant strain RA1062, Southern blot analysis was conducted as previously described [6]. Briefly, genomic DNA of the mutant strain RA1062 was extracted using the TIANamp Bacteria DNA kit (Tiangen), digested with the endonuclease *Xba*Ι, then subjected to sodium dodecyl sulfate polyacrylamide gel electrophoresis (SDS-PAGE) and transferred to a nitrocellulose membrane. After washing with saline sodium citrate, the membrane was immobilized for 2 h at 80 °C. PCR was performed to amplify the transposon-specific probe representing the 410-bp IS4351 fragment from the plasmid pEP4351 using the primer pair Tn4351-F/Tn4351-R (Table 1). The probe was generated and hybridization was conducted using the DIG DNA labeling and detection kit (Roche Diagnostics USA, Indianapolis, IN, USA), according to the manufacturer’s protocol. The plasmid pEP*4351* and genomic DNA of the WT strain CH3 were also subjected to hybridization analysis, for use as the positive and negative controls, respectively.

**Table 1:** **Strains, plasmids, and primers used in this study**

Strains, plasmids or primers	Characteristics	Source or references
Strains		
CH3	*Riemerella anatipestifer* serotype 1 strain	[6]
RA1062	Tn4351 insertion mutant of *Riemerella*. *anatipestifer* CH3, M949_RS01035:: Tn	This study
BW19851(pEP*4351*)	Plasmid pEP*4351* in BW19851, CmR	[6]
WJ4	*R. anatipestifer* WT strain, serotype 1	[6]
Yb2	*R. anatipestifer* WT strain, serotype 2	[6]
HXb2	*R. anatipestifer* WT strain, serotype 10	[6]
Primers		
16S rRNA F	5′-GAGCGGTAGAGTATCTTCGGATACT-3′	This study
16S rRNA R	5′-AATTCCTTTGAGTTTCAACCTTGCG-3′	This study
TN-1	5′-GGACCTACCTCATAG-3′	This study
IS4351-F	5′-TCAGAGTGAGAGAAAGGG-3′	This study
Tn4351-F	5′-TGGCACCTTTGTGGTTCTTAC-3′	This study
Tn4351-R	5′-GAGAGACAATGTCCCCCTTTC-3′	This study
Erm-F	5′-GCCCGAAATGTTCAAGTTGT-3′	This study
Erm-R	5′-CTTGACAACCACCCGACTTT-3′	This study
M949_RS10475-F	5′-CCAAACCATATGAACCATCCTGT-3′	This study
M949_RS10475-R	5′-GCATTATCTTCTGACAGGAGAGG-3′	This study
M949_RS01035F	5′-TATAAAGCCTACAATAGC-3′	This study
M949_RS01035R	5′-ATTAATTGAAGAGTTTGC-3′	This study
M949_RS01030-F	5′-TCAATTGCTGAATCCAAACGC-3′	This study
M949_RS01030-R	5′-TTCAGGCATTGTTGTGATGTC-3′	This study
RA ldh-F	5′-AGAGGAGCTTATCGGCATCA-3′	This study
RA ldh-R	5′-CTAGGGCTTCTGCCAATCTG-3′	This study

The nucleotide sequence surrounding the transposon insertion site was determined using inverse PCR, as described elsewhere [24]. Briefly, genomic DNA was digested with the restriction enzyme *Hin*dIII and then treated with T4 ligase, which resulted in the formation of circular molecules. Primer pairs specific for Tn4351 (primers TN-1 and IS4351-F) were used to amplify the sequences adjacent to the insertion site using the LA PCR kit (TaKaRa Biotechnology (Dalian) Co., Ltd., Dalian, China). The nucleotide sequence was compared to sequence in the National Center for Biotechnology Information database using the BLASTX program [25].

### Determination of the bacterial growth curves

The growth rates of the WT strain CH3 and the mutant strain RA1062 were determined and compared. Briefly, each bacterial strain was cultivated in TSB at 37 °C for 8 h with shaking. Equal amounts of each bacterial culture were then inoculated into 12-mL of fresh TSB medium at a ratio of 1:100 (v/v) and incubated at 37 °C, with shaking at 200 rpm, respectively. The bacterial growth rate was measured by counting the bacterial CFU at 2-h intervals for 16 h.

### Characterization of the bacterial phenotype

The phenotypes of the WT strain CH3 and its mutant strain RA1062 were determined by crystal violet staining, as previously described [42] with slight modifications. The bacterial cells were grown in TSB at 37 °C for 8 h, then harvested by centrifugation at 5000 rpm for 5 min, suspended in sterile PBS to a density of 10^3^ cells/mL, plated (0.1 mL) onto TSA, and incubated at 37 °C under an atmosphere of 5% CO_2_ for 36 h. The plates were then gently flooded with 5 mL of 0.5% crystal violet solution. After staining for 1 min, the excess stain was removed and the plates were examined immediately.

### LPS extraction, silver staining, and Western blot analysis

Lipopolysaccharide was extracted from WT strain CH3 and mutant strain RA1062 cells using an LPS extraction Kit (iNtRON Biotechnology, Boca Raton, FL, USA), according to the manufacturer’s instruction. Purified LPS was analyzed by SDS-PAGE. Gels were stained with silver to visualize the presence of LPS [26] and then counter stained with Coomassie blue to exclude contaminating proteins.

For Western blot analysis, the purified LPS was separated by SDS-PAGE and then transferred onto nitrocellulose membranes (Millipore, Billerica, MA, USA), which were blocked overnight at 4 °C in PBS containing 5% nonfat milk and then washed with PBST. The blots were incubated with anti-CH3 LPS as the primary mAb and then with an IRDYE680CW-conjugated donkey anti-mouse IgG polyclonal antibody (LI-COR Biosciences, Lincoln, NE, USA) for 1 h. The blots were visualized with an Odyssey two-color infrared imaging system (LI-COR Biosciences).

### Serum sensitivity assay

Bacterial susceptibility to normal duck sera was tested as described elsewhere [27], with slight modifications. Briefly, normal complement-sufficient duck sera (without anti-*R. anatipestifer* antibody) were collected from 18-old-day healthy Cherry Valley ducks, pooled, and filter-sterilized (0.22 μm). Pooled duck sera were diluted to 12.5%, 25%, and 50% (v/v) in pH 7.2 PBS. Then, 10-μL aliquots of bacterial suspension containing 10^6^ CFU in PBS was added to 190 μL of serial diluted duck sera, 100% heat-inactivated serum, or PBS alone, respectively. The reaction mixtures were incubated at 37 °C under an atmosphere of 5% CO_2_ for 30 min. Afterward, the bacterial culture was serial diluted by tenfold and the bacterial count in each sample was calculated by plating onto TSA plates at 37 °C under an atmosphere of 5% CO_2_ for 36 h. The experiment was performed in triplicate.

### Adhesion and invasion assays

Bacterial adhesion and invasion assays were performed using Vero cells (ATCC CCL-81) as described elsewhere [10]. Briefly, Vero cells (10^5^/well) were grown in 24-well tissue culture trays in Dulbecco’s modified Eagle medium (DMEM), containing 10% fetal bovine serum (Biowest, Nuaillé, France). Prior to infection, confluent monolayers of Vero cells were rinsed three times with sterile PBS and then infected with the WT strain CH3 or the mutant strain RA1062 at a multiplicity of infection of 100. Infected cells were incubated at 37 °C under an atmosphere of 5% CO_2_ for 1.5 h. Non-adherent bacteria were removed by washing three times with sterile PBS. The cell-adherent bacteria were enumerated following dispersion with PBS-0.1% trypsin, serial diluted tenfold, and spread onto TSA plates to estimate the number of bacteria (CFU) adhering to the cell monolayers. For the invasion assay, the cell culture, bacterial infection, and plating procedures were performed as described for the adherence assay. After bacterial infection, 1 mL of DMEM medium containing 100 μg/mL of gentamicin was added to each well and the plate was incubated at 37 °C under an atmosphere of 5% CO_2_ for additional 1 h to kill extracellular bacteria. All of samples were assayed in triplicate and the assay was conducted independently three times.

### Bacterial virulence determination

The median lethal dose (LD_50_) of the WT strain CH3 and the mutant strain RA1062 was determined using 18-day-old Cherry Valley ducks as described elsewhere [10]. The ducks were randomly divided into five groups (8 ducks/group), and inoculated intramuscularly with the appropriate bacterial strain at a dose of 10^6^, 10^7^, 10^8^, 10^9^, or 10^10^ CFU, respectively. Moribund ducks with clinical signs of diarrhoea, pyrexia, anorexia, stunted growth, respiratory signs, neurological abnormalities, or ocular signs were euthanized humanely with an intravenous injection of sodium pentobarbital at a dose of 120 mg/kg and counted as dead. On post-mortem, a yellow-white exudate and congestion can be seen throughout the body. Ducks were monitored daily for death rate for a period of 7 days post-infection to calculate LD_50_ value according to the improved Karber’s method [28]. The diagnosis was finally confirmed by bacterial isolation from liver, spleen and brain samples of dead ducks.

To evaluate bacterial survival in vivo, 18-day-old Cherry Valley ducks were divided randomly into two groups (6 ducks/group) and injected intramuscularly with 5 × 10^8^ CFU of the WT strain CH3 or the mutant strain RA1062. Blood samples were collected at 6, 12, 24, 48, and 72 h post-infection (hpi) (six ducks per group at each time point), diluted tenfold to appropriate concentrations, and plated in triplicate on TSA for bacterial counting.

### Illumina sequencing for RNA-Seq and differential gene expression analyses

The quantity and quality of total RNA were assessed using the Agilent RNA 6000 Nano Kit and the Agilent 2100 Bioanalyzer (Agilent Technologies, Santa Clara, CA, USA). Total RNA was treated with the Ribo-Zero Magnetic Gold Kit (Epicentre^®^ (an Illumina company), Madison, WI, USA) to remove ribosome RNA, then libraries were constructed using the TruSeq RNA Sample Prep Kit v2 (Illumina, Inc., San Diego, CA, USA) in accordance with the manufacturer’s instructions. The quality and quantity of the libraries were assessed by two methods: the distribution of the fragment size was checked using the Agilent 2100 bioanalyzer (Agilent DNA 1000 Reagents) and quantified using real-time qPCR (TaqMan Probe). The qualified libraries were amplified on cBot to generate the cluster on the flowcell (TruSeq PE Cluster Kit V3–cBot–HS, Illumina). Then, the amplified flowcell was sequenced via the paired-end method on the HiSeq2000 System (TruSeq SBS KIT-HS V3, Illumina), with a read length of 90 bp, which is the most common sequencing strategy.

The complete libraries were sequenced for 100 cycles on the Illumina HiSeq2000 system as described elsewhere [29]. Image analysis and base calling were performed using Solexa pipeline version 1.8 (Off-Line Base Caller software, version 1.8) [30]. Cleaned reads were aligned to the genome of *R. anatipestifer* strain CH3 using RNA Sequel software [31]. Transcript levels were calculated as reads per kilobase of cDNA per million fragments mapped. Differentially expressed genes were analyzed using Cufflinks software (version 2.1.1) with significance at a fold change cut-off of 2.0 [32] and considered statistically significant if the fold change was > 2.0 and false discovery rate of < 0.001.

### Real-time quantitative PCR (qPCR) analysis

qPCR was performed to confirm the transcriptional levels of differentially expressed genes obtained by RNA-Seq analysis. Gene-specific primers (Table 1) were designed using primer3 online software version 0.4.0 [33]. The expression levels of the l-lactate dehydrogenase encoding gene (ldh) were measured using the primer pair RA ldh-F/RA ldh-R (Table 1) and used as an internal control [34]. Total RNA was isolated from the WT strain CH3 and the mutant strain RA1062 using TRIzol reagent (Invitrogen Corporation, Carlsbad, CA, USA), according to the manufacturer’s instructions. All RNA samples were treated with the TURBO DNA-free kit (Ambion/Life Sciences, Grand Island, NY, USA) to remove DNA contamination. cDNA was synthesized using PrimeScript RT Master Mix (Takara). qPCR was conducted using Go Taq qPCR Master Mix (Promega, Fitchburg, WI, USA) with the following parameters: 95 °C for 2 min, 40 cycles of 95 °C for 15 s, 55 °C for 15 s and 68 °C for 20 s, followed by one cycle of 95 °C for 15 s, 60 °C for 15 s and 95 °C for 15 s. Reactions were performed in triplicate and run on the Mastercycler ep realplex4 apparatus (Eppendorf AG, Hamburg, Germany). Quantification of transcriptional level was calculated according to the 2^−ΔΔCt^ method.

### Vaccination and challenge assays

To determine whether the mutant strain RA1062 conveyed effective cross-protection among different *R. anatipestifer* strains, the inactivated RA1062 and CH3 vaccines were prepared using the mutant strain RA1062 and the WT strain CH3, respectively. Briefly, strains RA1062 and CH3 were cultured separately in TSB at 37 °C for 10 h with shaking. The bacterial culture was then adjusted to 10^10^ CFU/mL and inactivated with 0.4% (vol/vol) formalin at 37 °C for 16 h, respectively. The vaccine was made by blending 3 volume inactivated strains (RA1062 or CH3) and 7 volumes of Montanide ISA 70 VG adjuvant (Seppic Shanghai Special Chemical Corporation, Shanghai, China) according to the manufacturer’s protocol. Each duck was subcutaneously injected in the neck with 0.3 mL of the vaccine containing 10^9^ CFU bacterial cells of RA1062 or CH3 respectively. Cherry Valley ducks were divided randomly into three groups (24 ducks per group). The ducks in group 1 received two subcutaneous injections of the inactivated RA1062 vaccine, at a dose of 10^9^ CFU. Each duck in group 2 received two subcutaneous injections of the inactivated CH3 vaccine, and those in group 3 received two subcutaneous injections of saline in adjuvant for use as controls. At 14 days after the second immunization, eight ducks from each group were challenged with *R. anatipestifer* strain WJ4 (serotype 1), Yb2 (serotype 2), or HXb2 (serotype 10) at 10 LD_50_, respectively. Ducks were monitored daily for clinical symptoms and death until 7 days post-infection. The protection rate was calculated as follows: [1 − (no. of dead ducks per group/total no. of ducks per group)] × 100. The experiment was repeated for three times.

### Statistical analysis

All statistical analyses were performed using GraphPad Prism, version 5.0 for Windows software (GraphPad Software Inc., La Jolla, CA, USA). Adhesion and invasion assays, bacterial growth curves, bacterial loads in the blood of ducks, serum sensitivity assays, and RT-qPCR were two tailed, and a *p*-value of < 0.05 was considered statistically significant. Analysis of variance was used for comparisons of multiple groups.

## Results

### Identification of the mutant strain RA1062

**Figure 1:**
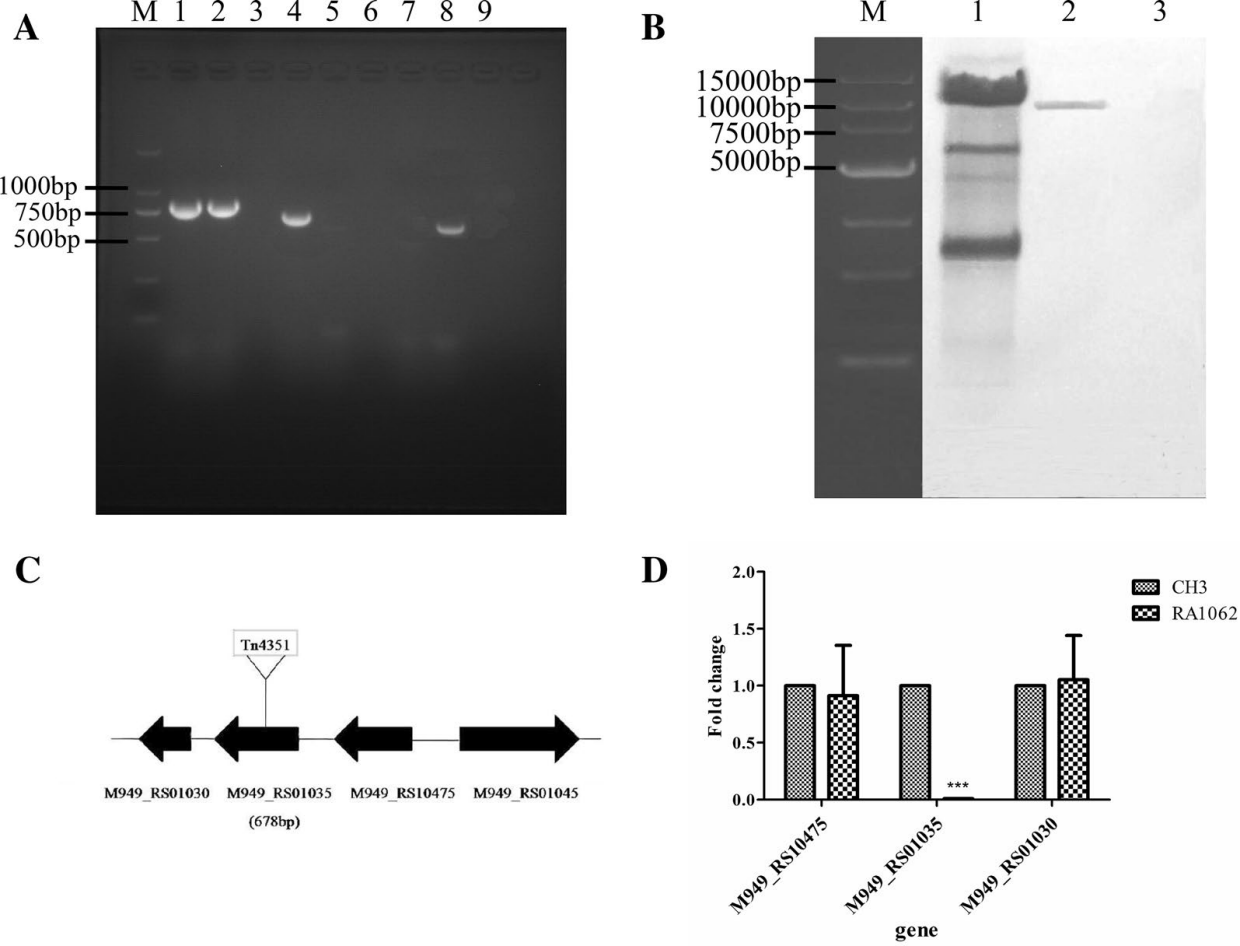
**Identification of the mutant strain RA1062. A** PCR amplification. M: Takara DL2000 marker; lanes 1–2: *R. anatipestifer* 16S rRNA was amplified from the WT strain CH3 (lane 1), the mutant strain RA1062 (lane 2), showing a 744-bp fragment of 16S rRNA; lanes 4–5: a 678-bp fragment of *M949_RS01035* was amplified from the WT strain CH3 (lane 4), but not the mutant strain RA1062 (lane 5); lanes 7–8: the 644-bp fragment of *erm* gene was not amplified from the WT strain CH3 (lane 7), but amplified from the mutant strain RA1062 (lane 8); lanes 3, 6 and 9: the avian pathogenic *E. coli* strain (APEC, CVCC1547), as negative controls. **B** Southern blot analysis of the transposon Tn4351 insertion. Lane 1, 10 μg of pEP*4351* digested with *Xba*I (positive control); Lane 2, 10 μg of chromosomal DNA from mutant strain RA1062 digested with *Xba*I; Lane 3, 10 μg of chromosomal DNA from the WT strain CH3 digested with *Xba*I (negative control). The digested sample was resolved on a 0.7% agarose gel and Southern blot analysis was performed using a TnDIG-labeled probe. **C** Schematic chart of Tn4351 insertion in RA1062 chromosome at 318 bp of the gene, which is 678 nucleotides in length. **D** qPCR analysis. The expression of the mRNAs were expressed as fold change and calculated using the comparative C_T_ (2^−∆∆CT^) method. Data were normalized to the housekeeping gene *ldh* and expressed as fold changes. The expression of *M949_RS01035* in the mutant strain RA1062 was disrupted. However, no change was shown for its upstream *M949_RS10475* gene and downstream *M949_RS01030* gene. Error bars represent standard deviations from three replicates (***p < 0.001).

The mutant strain RA1062, which lacked reactivity with the anti-CH3 LPS mAb, was obtained by screening the transposon library using an indirect ELISA and identified by PCR amplification using the primer pairs 16S rRNA F/16S rRNA R, Erm-F/Erm-R, and RA1062-F/RA1062-R. As shown in Figure 1A, a 744-bp fragment of 16S rRNA gene was amplified from the WT strain CH3 (lane 1) and the mutant strain RA 1062 (lane 2), a 678-bp fragment of the *M949_RS01035* gene was amplified from the WT strain CH3 (lane 4), but not from the mutant strain RA1062 due to the transposon insertion (lane 5), and a 644-bp fragment of the *erm* gene (contained in the Tn4351 transposon) was amplified from the mutant strain RA1062 (lane 8), but not from the WT strain CH3 (lane 7). The mutant strain RA1062 was confirmed to contain a single Tn4351 insertion in the chromosomal DNA by Southern blot analysis (Figure 1B, lane 2).

The transposon was inserted at nucleotide position 318 bp of the *M949_RS01035* gene, which is 678 nucleotides in length and encodes intramembrane metalloprotease of the CPBP (CAAX proteases and bacteriocin-processing enzymes) family, which consists of 225 amino acids (Figure 1C). BLAST analysis showed that *M949_RS01035* gene exists in *R. anatipestifer* serotype 1 strains CH3 and CH-1, as well as serotype 10 strain HXb2 (Additional file 1). qPCR analysis further confirmed that transcription of the *M949_RS01035* gene was abolished in the mutant strain RA1062 (Figure 1D). Further investigation showed that transcription of chromosomally upstream *M949_RS10475* gene, which encodes a hypothetical protein, and the downstream *M949_RS01030* gene, which encodes a “prevent host death” protein, had no significant changes, as compared with the WT strain CH3 (Figure 1D).

### Determination of bacterial growth curves

**Figure 2:**
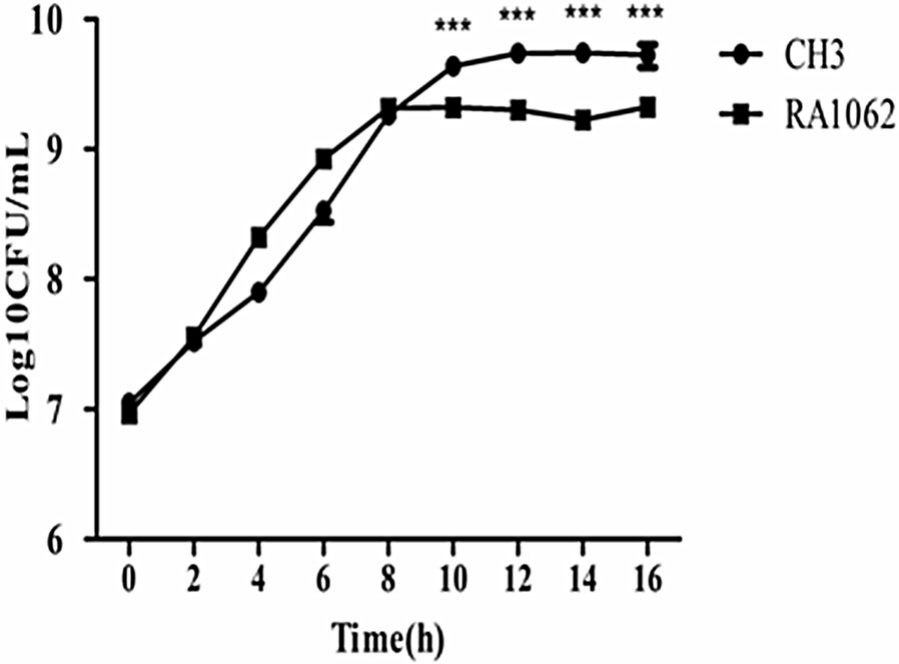
**Construction of bacterial growth curves.** Strains CH3 and RA1062 were grown in TSB at 37 °C with shaking, and the bacterial CFU were measured at 2-h intervals. The experiment was repeated three times and the data are presented as the mean ± standard deviation. Error bars represent standard deviations. Asterisks indicate statistically significant differences between groups (***p < 0.001).

Bacterial growth curves of the WT strain CH3 and the mutant strain RA1062 in TSB medium were constructed. Compared to that of the WT strain CH3, the growth rate of the mutant strain RA1062 was similar at the early growth stage, but then significantly decreased at the late growth stage. After 12 h in TSB, growth of the WT strain CH3 reached a plateau of 5.12 × 10^9^ CFU/mL, while the mutant strain RA1062 reached a plateau of 1.99 × 10^9^ CFU/mL after 8 h in TSB. The bacterial numbers of the mutant strain RA1062 at the plateau was about 2.79-fold less than that of the WT strain CH3 (Figure 2).

### Bacterial phenotype characterization by crystal violet staining

**Figure 3:**
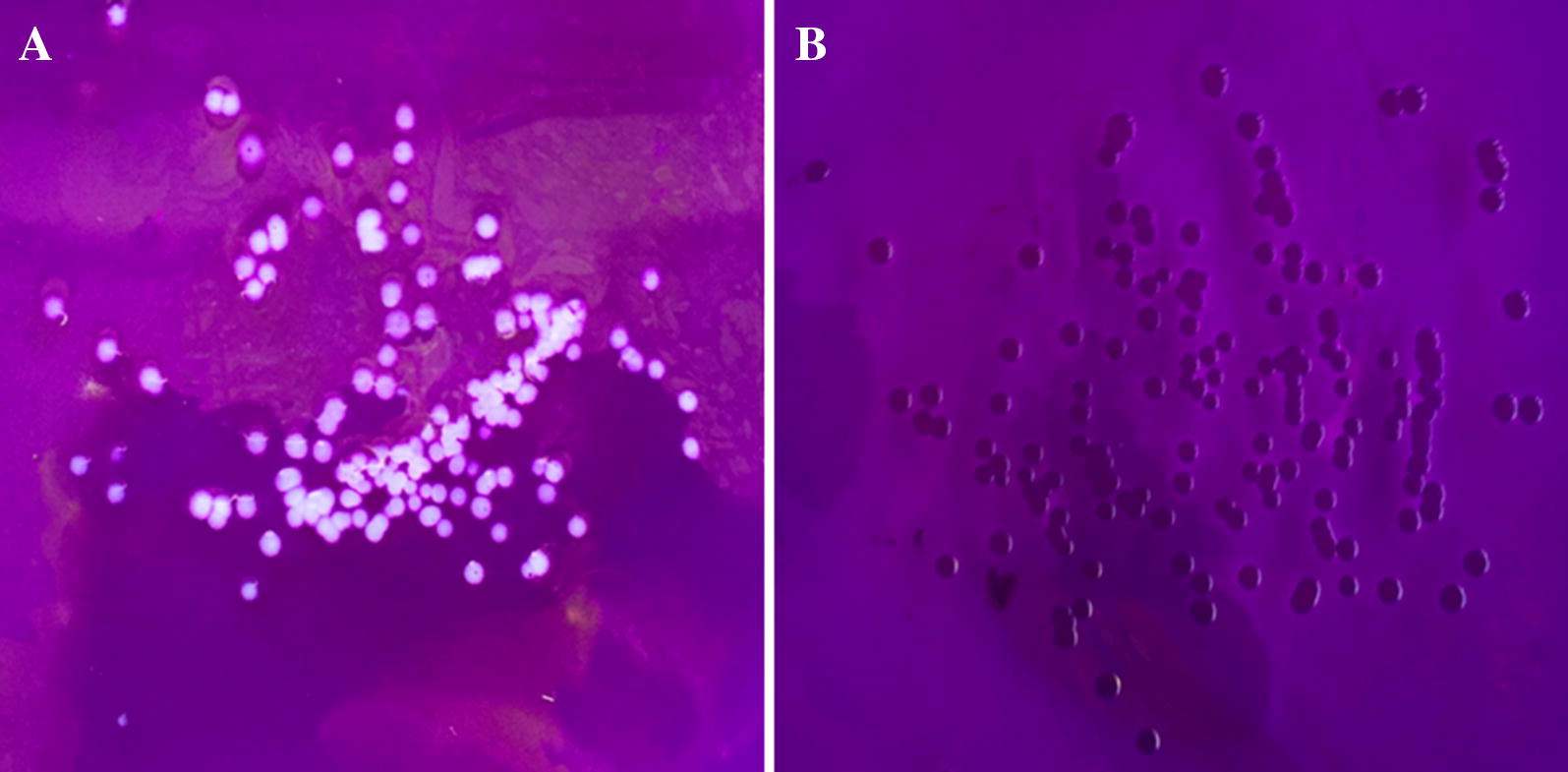
**Crystal violet staining.** Crystal violet staining was used to differentiate the bacterial phenotypes. **A** CH3 strain; **B** RA1062 mutant. Mutant strain RA1062 was stained by crystal violet.

Crystal violet staining was used to characterize the bacterial phenotypes of the WT strain CH3 and the mutant strain RA1062. The result indicated that the WT strain CH3 was not stained by crystal violet (Figure 3A), while the mutant strain RA1062 was (Figure 3B), suggesting a change in its phenotype of the mutant strain RA1062.

### Analysis of the bacterial LPS by silver staining and Western blot

**Figure 4:**
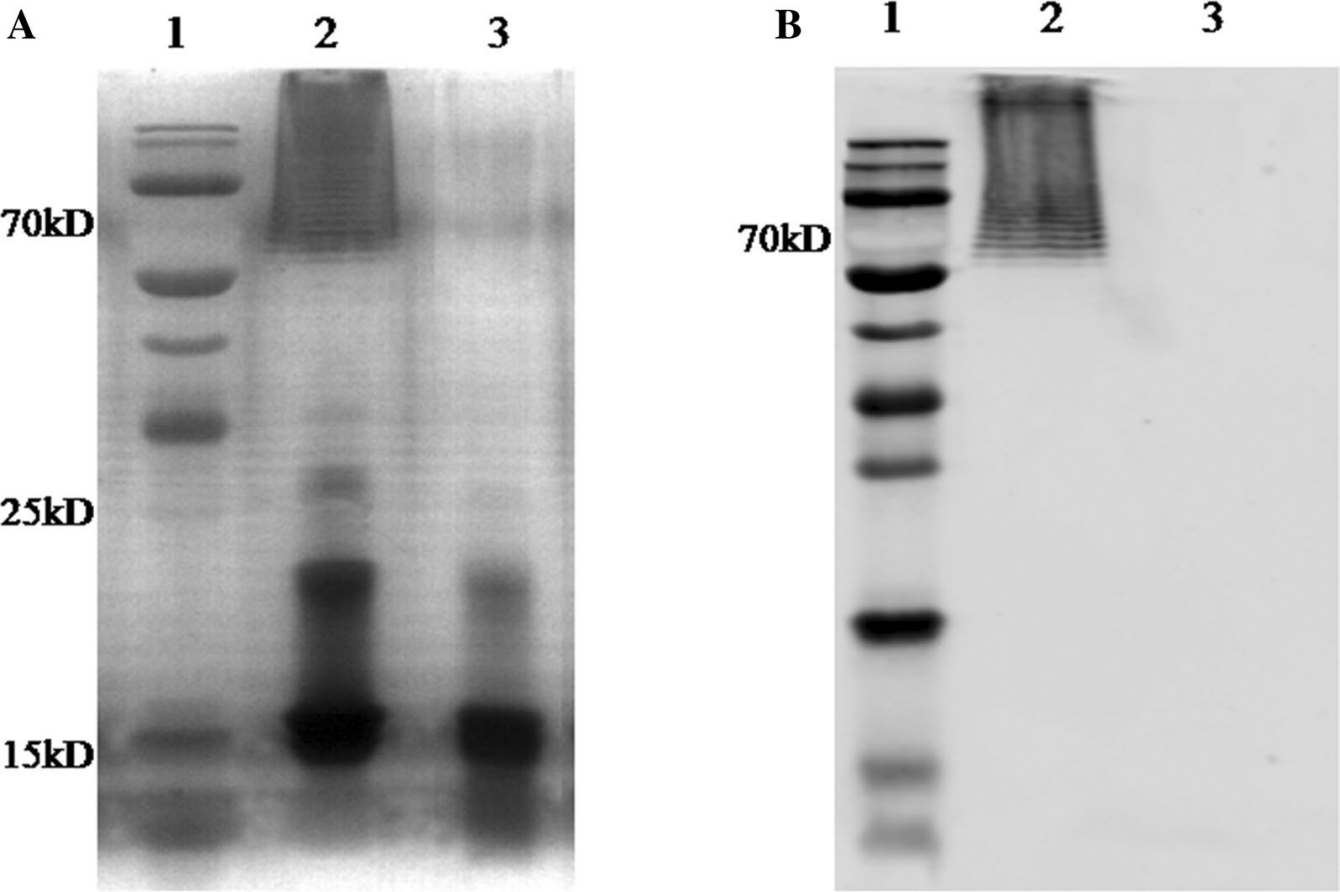
**Detection of bacterial LPS.** LPS samples were prepared using the LPS extraction kit, according to the manufacturer’s instruction, and subjected to SDS-PAGE on a 15% polyacrylamide gel. **A** Silver staining. **B** Western blot analysis. Lane 1: molecular weight marker; lane 2: the WT strain CH3; Lane 3: the mutant strain RA1062.

LPS was purified from the WT strain CH3 and the mutant strain RA1062, then subjected to SDS-PAGE followed by silver staining and Western blot analysis. As shown in Figure 4A, LPS purified from the WT strain CH3 displayed a ladder-like pattern around 70 kDa (lane 2), while the ladder-like pattern was deficient in the mutant strain RA1062 LPS (lane 3) in the silver staining. Western blot analysis using the anti-CH3 LPS mAb detected a ladder-like pattern of O-antigen repeats in the CH3 strain (lane 2), but the pattern was defective in the mutant strain RA 1062 (Figure 4B). These results suggest that the *R. anatipestifer M949_RS01035* gene is involved in LPS O-antigen biosynthesis.

### Adhesion and invasion assays

**Figure 5:**
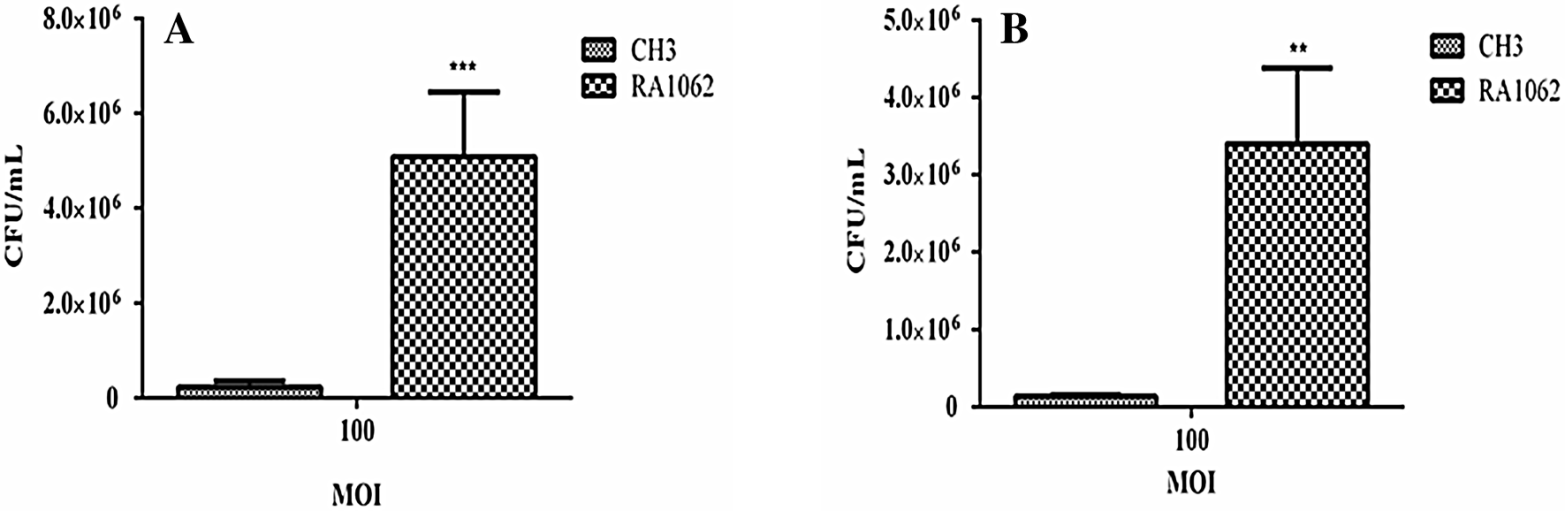
**Bacterial adherence and invasion assays.** Strains CH3 and RA1062 were tested on Vero cells. **A** Adherence assay; **B** Invasion assay. The data represent the number of bacteria bound to or invaded into Vero cells in each well of a 24-well plate. The error bars represent mean ± standard deviations from three independent experiments (**p < 0.01; ***p < 0.001). The adherence and invasion capacities of the RA1062 mutant were significantly increased in comparison with its WT strain CH3.

To determine the role of the *M949_RS01035* gene in bacterial adherence and invasion, we compared bacterial adhesion and invasion abilities of the WT strain CH3 and the mutant RA1062 on Vero cells. The results showed that the mutant strain RA1062 displayed approximately 21.92-fold enhanced adherence ability (Figure 5A) and 25.18-fold increased invasion capacity (Figure 5B) in comparison with those of the WT strain CH3 (*p *< 0.001).

### The mutant strain RA1062 was more sensitive to normal duck sera

**Figure 6:**
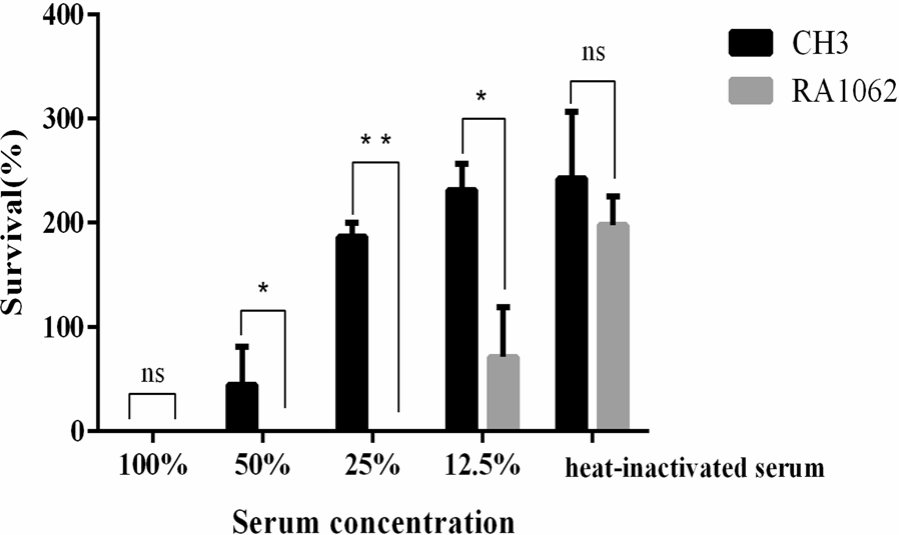
**Bacterial serum resistance assay.** Bacteria were incubated with normal duck sera at different dilutions at 37 °C and enumerated after 30 min of incubation. Three experimental replicates were performed and the data presented as the mean ± standard deviation. Resistance of RA1062 to normal duck sera was significant decreased, as compared to CH3 (*p < 0.05; **p < 0.01). The survival rate (%) was calculated as follows: (bacterial CFU with sera treatment/bacterial CFU with PBS treatment) × 100.

To determine whether the *M949_RS01035* gene is involved in serum resistance of the WT strain CH3, we compared the abilities of the WT strain CH3 and the mutant strain RA1062 to resist the complement-mediated killing. The results showed that 25% diluted serum was effective in killing the mutant strain RA1062, but not the WT strain CH3, indicating that the mutant strain RA1062 was more sensitive to normal duck sera than the WT strain CH3 (Figure 6).

### Determination of bacterial virulence

**Figure 7:**
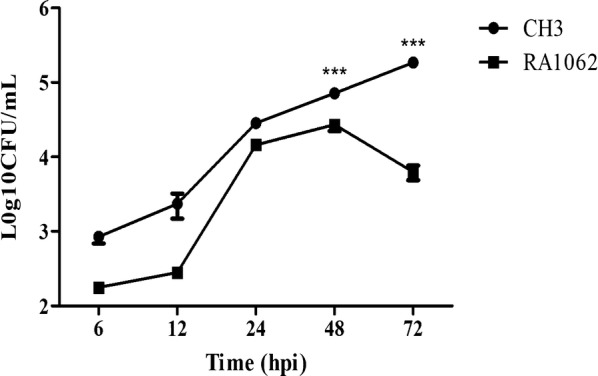
**Determination of bacterial virulence.** Bacterial loads in the blood of ducks infected with CH3 or RA1062 at 6, 12, 24, 48, and 72 hpi. Three experimental replicates were performed and the data presented in the figure were from one representative experiment. The error bars represent means ± standard deviations from six ducks. Asterisks indicate statistically significant differences between groups (***p < 0.001).

Bacterial virulence was evaluated based on LD_50_ determination using 18-day-old Cherry Valley ducks. The LD_50_ of the mutant strain RA1062 was 2.74 × 10^10^ CFU, which was more than 365-fold greater attenuated virulence than that of the WT strain CH3 (7.50 × 10^7^ CFU). In addition, to confirm the role of the *M949_RS01035* gene in systemic invasion and dissemination, bacterial loading in the blood of ducks infected with the WT strain and the mutant strain RA1062 was conducted. As shown in Figure 7, the bacterial loads in the blood of ducks infected with the WT strain CH3 continued to increase up to 72 hpi, while that in the blood of ducks infected with the mutant strain RA1062 decreased from 48 hpi, indicating a significant decrease in comparison with ducks infected with the WT strain CH3 at 48 and 72 hpi (*p *< 0.001). This experimental result further confirmed the attenuated virulence of the mutant strain RA1062.

### Identification of differentially expressed genes

Strand-specific Illumina RNA-Seq analysis was used to identify differentially expressed genes between the WT strain CH3 and the mutant strain RA1062. In total, 12 genes were up-regulated and nine were down-regulated in the mutant strain RA1062 in comparison to the WT strain CH3 (Table 2), which were differentially expressed over fivefold based on RNA-Seq analysis. qPCR further confirmed that the *M949_RS07300* and *M949_RS04680* genes were up-regulated, and the *M949_RS07580* and *M949_RS03025* genes were down-regulated by more than fivefold at the transcriptional level. The proteins encoded by the *M949_RS07300*, *M949_RS04680*, and *M949_RS03025* genes were annotated as TonB-dependent receptors. The coding product of the *M949_RS07580* gene was annotated as the polysaccharide biosynthesis protein CapD, which is responsible for bacterial capsule polysaccharide biosynthesis. These results indicated that the *M949_RS01035* gene regulated several genes responsible for bacterial polysaccharide biosynthesis.

**Table 2:** **qPCR verification of differentially expressed genes in the mutant strain RA1062**

Gene locus^a^	Description of genes	Fold-change
M949_RS07300	TonB-dependent receptor	7.80
M949_RS04680	TonB-dependent receptor	7.57
M949_RS02830	TonB-dependent receptor	4.58
M949_RS02835	Hypothetical protein	3.37
M949_RS09405	Transcriptional regulator	2.34
M949_RS10455	DNA-binding protein	2.21
M949_RS10515	Hypothetical protein	1.85
M949_RS02445	Leucine-rich repeat-containing protein	1.73
M949_RS10510	S41 family peptidase	1.50
M949_RS08410	Hypothetical protein	1.08
M949_RS06030	Hypothetical protein	0.93
M949_RS02320	Hypothetical protein	0.92
M949_RS05620	Hypothetical protein	0.90
M949_RS02480	Hypothetical protein	0.86
M949_RS02475	Hypothetical protein	0.83
M949_RS04495	Phosphate sodium symporter	0.68
M949_RS10460	Hypothetical protein	0.65
M949_RS10465	Hypothetical protein	0.53
M949_RS05615	DNA-binding protein	0.42
M949_RS03025	TonB-dependent receptor	0.16
M949_RS07580	Polysaccharide biosynthesis protein CapD	0.03

^a^Based on the genome of *R. anatipestifer* strain CH3 (Accession Number: CP006649).

### Cross-protection experiment

To investigate the cross-protection against *R. anatipestifer* serotypes 1, 2, and 10, the RA1062-vaccinated ducks were challenged with virulent *R. anatipestifer* strains WJ4 (serotype 1), Yb2 (serotype 2), and HXb2 (serotype 10) at 10 LD_50_, respectively. Protection from challenge with strains WJ4, Yb2, and HXb2 was greater in RA1062-vaccinated ducks than those vaccinated with CH3. In contrast, all non-vaccinated ducks were dead within 7 days post-challenge. Three experimental replicates showed similar cross-protection results (Table 3). These results indicated that RA1062 provided cross-protection against challenge with *R. anatipestifer* serotypes 1, 2, and 10, which further confirmed its altered antigenicity.

**Table 3:** **Cross-protection experiment**

Immunization	Challenge strains^a^	Protection rate (%)^b^	Geomean of three protection rates (%)	SD of three protection rates
Inactivated RA1062 vaccine	WJ4	100.0; 100.0; 100.0	100.00	0.00
	Yb2	87.5; 75.0; 75.0	78.95	7.22
	HXb2	100.0; 100.0; 100.0	100.00	0.00
Inactivated CH3 vaccine	WJ4	87.5; 100.0; 100.0	95.65	7.22
	Yb2	25.0; 25.0; 25.0	25.00	0.00
	HXb2	50.0; 50.0; 50.0	50.00	0.00
Non-vaccinated	WJ4	0; 0; 0	0.00	0.00
	Yb2	0; 0; 0	0.00	0.00
	HXb2	0; 0; 0	0.00	0.00

^a^Challenge dose was 10 LD_50_ for each strain.

^b^The protection rate was calculated as follows: [1 − (no. of dead ducks per group/total no. of ducks per group)] × 100. The protection rates for three experimental repeats were shown in the table and separated with semicolon.

## Discussion

This study was initially designed to discover genes of *R. anatipestifer* strain CH3 involved in LPS biosynthesis and to explore potential use of the mutant for development of a cross-protective vaccine. The mutant strain RA1062 was obtained by screening the random Tn4351 transposon mutant library with an indirect ELISA, which lacked reactivity with the anti-CH3 LPS mAb. Further investigations revealed that the phenotype of the mutant strain was altered with a decreased growth rate in TSB, increased sensitivity to normal duck serum, and increased bacterial adhesion and invasion capacities on Vero cells, as compared to the WT strain CH3. Sequence analysis revealed that the transposon was inserted into the *M949_RS01035* gene of strain CH3 at 318 bp.

The protein encoded by the *M949_RS01035* gene is annotated as an intramembrane metalloprotease of the CPBP (CAAX proteases and bacteriocin-processing enzymes) family, which exhibited a high degree of similarity with the Abi family proteins of *Lactobacillus plantarum*. The members of the Abi protein family are reportedly involved in bacteriocin self-immunity in streptococci [35]. A BLAST search indicated that the *M949_RS01035* gene exists in *R. anatipestifer* serotypes 1 strains CH3 and CH-1, as well as serotype 10 strain HXb2. Silver staining indicated a marked deficiency of the ladder-like pattern in RA1062 mutant LPS, as compared to CH3 LPS. Western-blot analysis further confirmed that the modified LPS was less reactive with the anti-CH3 LPS mAb, suggesting that the *M949_RS01035* gene is associated with the LPS O-antigen biosynthesis.

It has been reported that the O-antigen side-chains are main components of LPS molecules of *E. coli* and *Salmonella typhimurium* involved in serum resistance by virtue of their anti-complement activities [36]. In the present study, the mutant strain RA1062 exhibited increased sensitivity to normal duck serum. Therefore, we speculated that the higher sensitivity of the mutant strain RA1062 to normal duck serum may due to a defect to the O-antigen, leading to easier bacteria killing by serum. It is also well established that the O-antigen plays a significant role in bacterial virulence [37]. Animal experiments further demonstrated that the virulence of mutant strain RA1062 was significantly decreased, as compared to the WT strain CH3. These experimental data demonstrate that bacterial O-antigen is not only involved in bacterial resistance to complement-mediated killing, but also an important virulence factor. It is established that bacterial adherence to the cell surface is the first step in the pathogenesis of microbial infections and functions as a critical determinant in colonization [10]. In this study, we found that both adherence and invasion capacities of the mutant strain RA1062 to Vero cells were significantly increased, as compared to those of the WT strain CH3. This phenomenon is consistent with rough mutants of *Brucella abortus*, which are taken up in greater numbers by macrophages than the smooth parental strains [38]. The enhanced ability of the mutant strain RA1062 to adhere to and invade Vero cells may also be dependent on the rough LPS phenotype, which employs different adherence and invasion mechanisms, as described for *B. abortus* [39].

Various antibiotics are currently used to prevent and control *R. anatipestifer* infection in ducks. However, the emergence of drug-resistant and multi-drug resistant strains due to the overuse of antibiotics has become a major barrier in *R. anatipestifer* treatment [2, 40]. Therefore, immunization of ducks with vaccine provides a valuable alternative to the use of antibiotics. Our study showed that ducks immunized with the mutant strain RA1062 were protected from challenge with *R. anatipestifer* strains WJ4 (serotype 1), Yb2 (serotype 2), and HXb2 (serotype 10) at ≥ 8/8, 6/8 and 8/8, respectively, suggesting that the RA1062 strain is an effective cross-protective vaccine candidate. A previous study reported that conserved LPS epitope(s) exist among different species and genera of non-enteric, Gram-negative, human pathogens [41]. The ability of the mutant strain RA1062 to provide broad cross-protection may be due to the deficiency of LPS O-antigen, which leads to the presence of some conserved LPS epitopes. Another possibility is that some cross-immunogenic components of the *R. anatipestifer* outer membrane, such as outer membrane proteins, are exposed at the bacterial surface of the mutant strain RA1062. However, based on current experiments, the mechanism of the cross-protection induced by the mutant strain RA1062 vaccination remains unclear, thus further work is needed to clarify the nature of the cross-protection.

RNA-Seq has rapidly become the method of choice for the study of differential gene expression, as it enables the investigation and comparison of gene expression levels at unprecedented resolution [42]. In our current RNA-Seq study, we found that 12 genes were up-regulated and nine were down-regulated by over fivefold in the mutant strain RA1062. qPCR verification further confirmed that two genes were up-regulated and two were down-regulated by over 5-fold. The proteins encoded by two up-regulated genes (*M949_RS07300* and *M949_RS04680*) and one down–regulated gene (*M949_RS03025*) are TonB-dependent receptors. TonB-dependent receptors are members of a family of beta-barrel proteins located on the outer membrane of Gram-negative bacteria, which are known to be mainly involved in iron or vitamin B12 uptake [43]. The crystallographic structures of two TonB-dependent receptors (FhuA and FepA) have recently been determined [44]. A recent study has identified the TonB-dependent receptor TbdR1 in *R. anatipestifer* strain CH3, which is a cross-immunogenic protein among strains with different serotypes [45]. Further investigation demonstrated that TbdR1 had a crucial role in the process of iron acquisition and complicated pathogenesis of *R. anatipestifer* strain CH3 [46]. However, how these TonB-dependent receptors are regulated by the *M949_RS01035* gene remains unknown, thus further studies are needed to elucidate the nature of this phenomenon. The *M949_RS07580* gene, which encodes the polysaccharide biosynthesis protein CapD, was significantly down-regulated by over 30-fold, which was consistent with the findings of our previous study [19]. It has been established that the polysaccharide biosynthesis protein CapD is a novel pathogenicity-associated determinant that is involved in the serum-resistance ability of *H. parasuis* and *E. faecium* [47, 48]. Protein sequence analysis indicated that there is a NAD-binding domain in the polysaccharide biosynthesis protein CapD, which is reportedly associated with LPS O-antigen biosynthesis [49]. Additionally, the polysaccharide biosynthesis protein CapD is responsible for the biosynthesis of type 1 capsular polysaccharides in *Staphylococcus aureus* [50]. Previous work has demonstrated that Group 1 and 4 capsules are related to LPS O-antigens [51]. Based on our current knowledge, we speculated that the loss of the LPS O-antigen in the mutant strain RA1062 may be associated with the down-regulation of the *M949_RS07580* gene, although further investigations are needed to elucidate the underlying mechanism.

In conclusion, we demonstrated that the *M949_RS01035* gene is involved in bacterial phenotype, virulence, and gene regulation in *R. anatipestifer.* The mutant strain RA1062 could be used as a novel cross-protective vaccine candidate for further vaccine development.

## Additional file

**Additional file 1.** Sequence analysis of *M949_RS01035* gene in *R. anatipestifer.*

## Abbreviations

LPS: lipopolysaccharide
mAb: monoclonal antibody
ELISA: enzyme-linked immunosorbent assay
PCR: polymerase chain reaction
CFU: colony forming unit
SDS-PAGE: sodium dodecyl sulfate polyacrylamide gel electrophoresis
LD_50_: median lethal doses
