# Supplementary figures and images for: The Riemerella anatipestifer M949_RS01035 gene is involved in bacterial lipopolysaccharide biosynthesis
Source: Vet Res. 2018 Sep 17;49:93. doi: 10.1186/s13567-018-0589-8 (PMC6142336; doi:10.1186/s13567-018-0589-8)

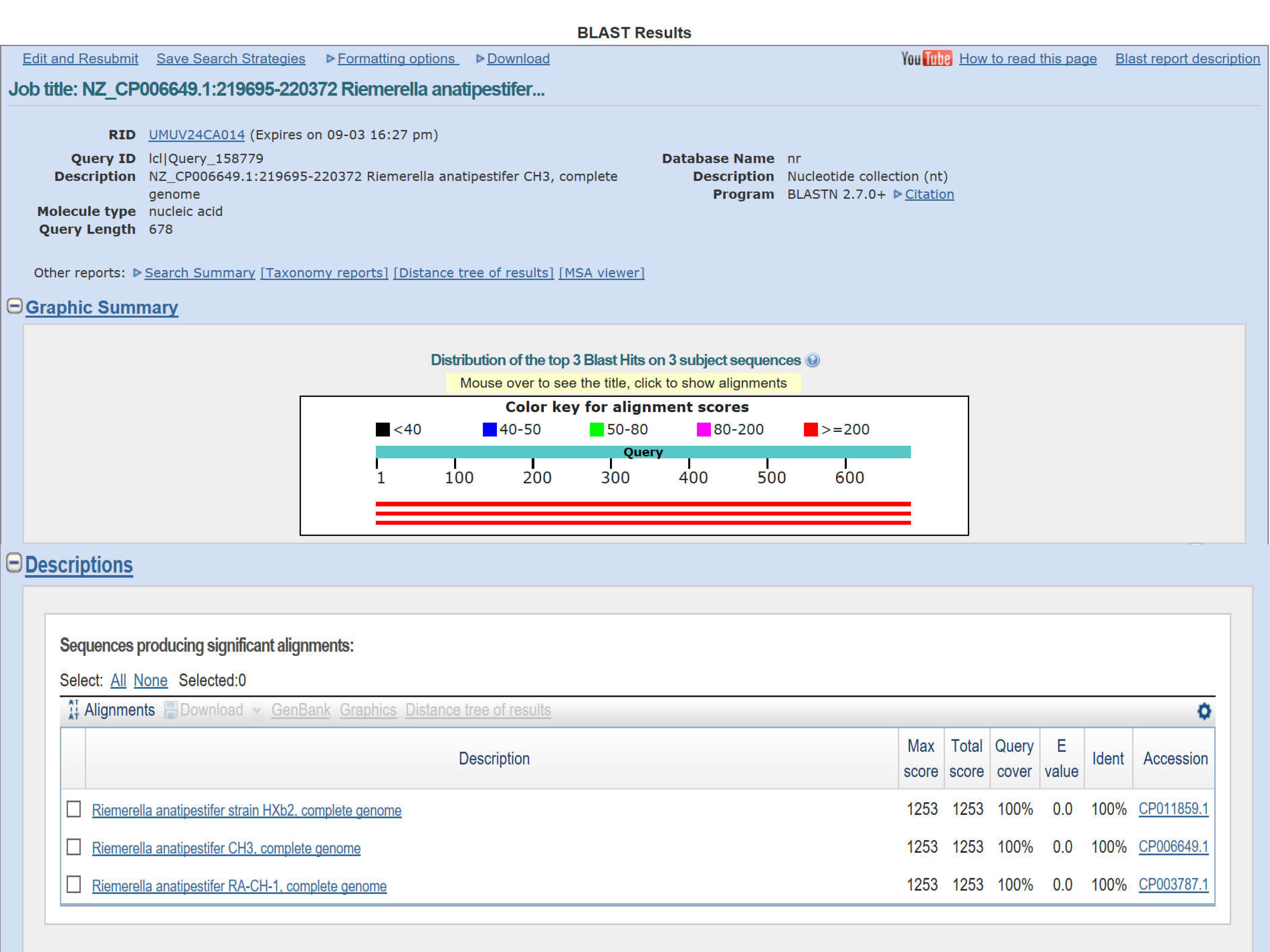

Supplement: Supplementary file 1 — Additional file 1. Sequence analysis of M949_RS01035 gene in R. anatipestifer. [file 13567_2018_589_MOESM1_ESM.tif]
